# DISC1 and reelin interact to alter cognition, inhibition, and neurogenesis in a novel mouse model of schizophrenia

**DOI:** 10.3389/fncel.2023.1321632

**Published:** 2024-01-12

**Authors:** Heather L. Mahoney, Charissa A. Bloom, Hannah S. Justin, Bianca M. Capraro, Christopher Morris, David Gonzalez, Emily Sandefur, John Faulkner, Samantha Reiss, Angel Valladares, Angie Ocampo, Bethany Carter, April L. Lussier, Linh Phuong Dinh, Edwin Weeber, Joshua Gamsby, Danielle Gulick

**Affiliations:** Department of Molecular Medicine, Morsani College of Medicine, University of South Florida, Tampa, FL, United States

**Keywords:** schizophrenia, DISC1, reelin, neurogenesis, mouse model, cognition

## Abstract

The etiology of schizophrenia (SCZ) is multifactorial, and depending on a host of genetic and environmental factors. Two putative SCZ susceptibility genes, Disrupted-in-Schizophrenia-1 (DISC1) and reelin (RELN), interact at a molecular level, suggesting that combined disruption of both may lead to an intensified SCZ phenotype. To examine this gene–gene interaction, we produced a double mutant mouse line. Mice with heterozygous RELN haploinsufficiency were crossed with mice expressing dominant-negative c-terminal truncated human DISC1 to produce offspring with both mutations (HRM/DISC1 mice). We used an array of behavioral tests to generate a behavioral phenotype for these mice, then examined the prefrontal cortex and hippocampus using western blotting and immunohistochemistry to probe for SCZ-relevant molecular and cellular alterations. Compared to wild-type controls, HRM/DISC1 mice demonstrated impaired pre-pulse inhibition, altered cognition, and decreased activity. Diazepam failed to rescue anxiety-like behaviors, paradoxically increasing activity in HRM/DISC1 mice. At a cellular level, we found increased α1-subunit containing GABA receptors in the prefrontal cortex, and a reduction in fast-spiking parvalbumin positive neurons. Maturation of adult-born neurons in the hippocampus was also altered in HRM/DISC1 mice. While there was no difference in the total number proliferating cells, more of these cells were in immature stages of development. Homozygous DISC1 mutation combined with RELN haploinsufficiency produces a complex phenotype with neuropsychiatric characteristics relevant to SCZ and related disorders, expanding our understanding of how multiple genetic susceptibility factors might interact to influence the variable presentation of these disorders.

## 1 Introduction

Schizophrenia (SCZ) is a devastating disorder, with an estimated US economic burden of $155.7 billion (Cloutier et al., 2016). It directly affects 1% of the population, and has a far-reaching impact on family, community, and caregivers. SCZ is characterized by positive (hallucinations), negative (impaired social abilities, reduced affect), and cognitive symptoms (Schuitz and Andreasen, 1999), including working memory deficits (Kar and Jain, 2016). This neurodevelopmental disorder usually manifests in early adulthood and results from genetic and environmental effects, as twin and family studies demonstrate 60%–90% disease liability from genetic factors (Neale and Sklar, 2015). Underlying pathology include alterations in the GABA (γ-aminobutyric acid) system, including decreased GAD67 (67-kilodalton isoform of glutamic acid decarboxylase) particularly in parvalbumin-expressing GABAergic neurons in the dorsolateral prefrontal cortex (PFC) (Lewis et al., 2005). Reductions in gray matter, dendritic spine density, and arborization are also observed (Moyer et al., 2015). While an extensive body of literature examines individual risk factors for SCZ, comparatively few address the potential effects of gene–gene interactions. Here we evaluated the validity of a novel gene–gene interaction mouse model of SCZ using mice with mutations in genes associated with schizophrenia, DISC1 and RELN.

The extracellular matrix glycoprotein reelin, encoded by the RELN gene, is essential in both development and adulthood. Brains from human SCZ patients have reduced reelin expression in multiple areas, including the hippocampus (HPC) and PFC (Impagnatiello et al., 1998; Eastwood and Harrison, 2006; Fatemi et al., 2008). Variations in RELN are positively associated with SCZ (Fromer et al., 2014; Zhou et al., 2016), and are also risk factors when combined with variations in other genes like brain-derived neurotrophic factor (Hall et al., 2007). During development, Cajal–Retzius cells secrete reelin in the cortex and hippocampus, which is essential for neuronal migration and dendritogenesis (Trommsdorff et al., 1999; Niu et al., 2004). In adults, reelin is essential for learning and memory, synaptic plasticity, and neurogenesis in adult brains (Pujadas et al., 2010; Lussier et al., 2011). Post-development, reelin is expressed by somatostatin-expressing neurons, as well as parvalbumin-expressing GABAergic interneurons in the cortex and hippocampus, which are reactive to prenatal immune activation and adolescent stress, key environmental factors in the development of SCZ (Giovanoli et al., 2014).

Reelin expression is reduced by approximately 50% in the heterozygous reeler mouse (HRM), a reduction similar in magnitude to the postmortem psychotic brain (Impagnatiello et al., 1998). These mice have deficits in prepulse inhibition, cognition, and executive function; reduced GAD67; reduced dendritic spine density and arborization in the cortex and hippocampus; and deficits in long-term potentiation (LTP) (Tueting et al., 2006).

DISC1 encodes the protein Disrupted-in-Schizophrenia-1 (DISC1), which – when truncated – has been linked to SCZ (Pletnikov et al., 2008). DISC1 complexes with other neurodevelopmental proteins to enable signal transduction and neurite outgrowth (Ozeki et al., 2003; Newburn et al., 2011). DISC1 is highly expressed in the forebrain during development, corresponding with RELN’s expression pattern and the timeline of SCZ susceptibility.

Disrupted-in-Schizophrenia-1 transgenic mice express a truncated human DISC1 gene under control of the tetracycline-controlled transactivator (tTa) driven by the Calcium/Calmodulin Dependent Protein Kinase II Alpha (CAMKIIα) promoter, which localizes the mutant DISC1 protein to the forebrain. Expression is limited in astrocytes or microglia, and is localized to neurons in the olfactory bulbs, frontal cortex, and hippocampal pyramidal and granule cells (Pletnikov et al., 2008).

**FIGURE 1:**
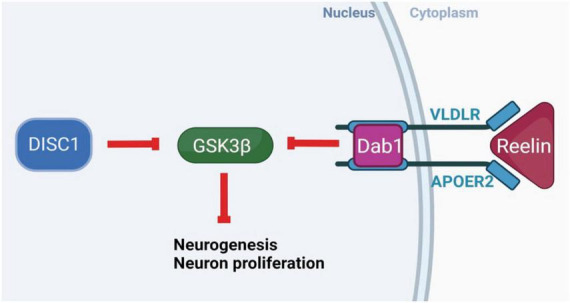
Molecular interactions of DISC1 and reelin. Intracellular DISC1 and extracellular reelin show parallel neurobiological effects, contributing to the pathways that control neurogenesis, neuronal migration, synaptic plasticity, and substrates of learning and memory. These genes share a common molecular pathway through which they normally act to phosphorylate and inhibit GSK3β, DISC1 directly, and reelin through receptor binding and the Dab1 intracellular signaling pathway. The inhibition of GSK3β disinhibits downstream gene expression to increase neurogenesis, synaptogenesis, neuron growth and survival. (Created with BioRender.com.)

Reelin and DISC1 share pathways regulating neurogenesis and neuronal migration. DISC1 complexes with lissencephaly-1 protein (LIS1) and nuclear distribution element-like protein (NUDEL), and may interact with reelin receptors and downstream effectors, influencing neuronal migration (Dobyns et al., 1993; Assadi et al., 2003, 2008; Figure 1). Reelin and DISC1 signaling pathways converge on the serine-threonine kinase glycogen synthase kinase 3β (GSK3β), a downstream effector in a number of signaling pathways implicated in SCZ (Jossin and Goffinet, 2007; Wu et al., 2013). An RNA-sequencing profile of hiPSCs (human-induced pluripotent stem cells) from a population of SCZ patients identified a large number of differentially expressed genes in the phosphoinositide 3-kinase/glycogen synthase kinase 3 (PI3K/GSK3) signaling pathway, including serum-glucocorticoid kinase 1 (SGK1), which is an inhibitor of GSK3β found to be overexpressed in SCZ patients (Stertz et al., 2021) Intriguingly, reelin is also thought to regulate dendritic outgrowth in the hippocampus via the mammalian target of rapamycin (mTOR) pathway independently of GSK3β (Jossin and Goffinet, 2007), while DISC1 interacts directly with GSK3β to influence neurogenesis, differentiation, neuron growth and survival, and (via β-catenin) dendritic arborization (Gao et al., 2007; Wu et al., 2013). There is a complex web of direct and indirect interactions between reelin, DISC1, and pathways associated with SCZ via genome-wide association studies, including the Wnt and mTOR pathways (Network and Pathway Analysis Subgroup of Psychiatric Genomics Consortium, 2015). These interactions encourage the hypothesis that the combined disruption of reelin and DISC1 genes might interact to produce widespread SCZ-like changes, particularly in neurogenesis.

Therefore, we crossed mice from HRM, DISC1, and Camk2a-tTa tet-off lines to produce HRM/DISC1 mice with a combined disruption of both reelin and DISC1. We hypothesized that this would result in a more severe SCZ-like phenotype than either mutation alone, including behavioral and learning deficits, and altered neurogenesis characterized by aberrant maturation of adult-born neurons in the hippocampus.

## 2 Results

Our first goal was to generate a phenotypic profile of HRM/DISC1 mice, both to determine whether they display SCZ-associated phenotypes, and to ensure that there were no gross behavioral abnormalities that might confound interpretation of other behavioral results. It is essential not only to investigate how this phenotype compares to wild-type (WT) controls, but also categorize differences between this new gene–gene interaction model and the single mutation HRM and mutant human DISC1 models that have already been well characterized. To this end, we performed our behavioral tests on adult male mice, 2–6 months of age, in four groups: WT, HRM, DISC1, and HRM/DISC1.

### 2.1 Altered activity, thigmotaxis, and prepulse inhibition in HRM/DISC1 mice

**FIGURE 2:**
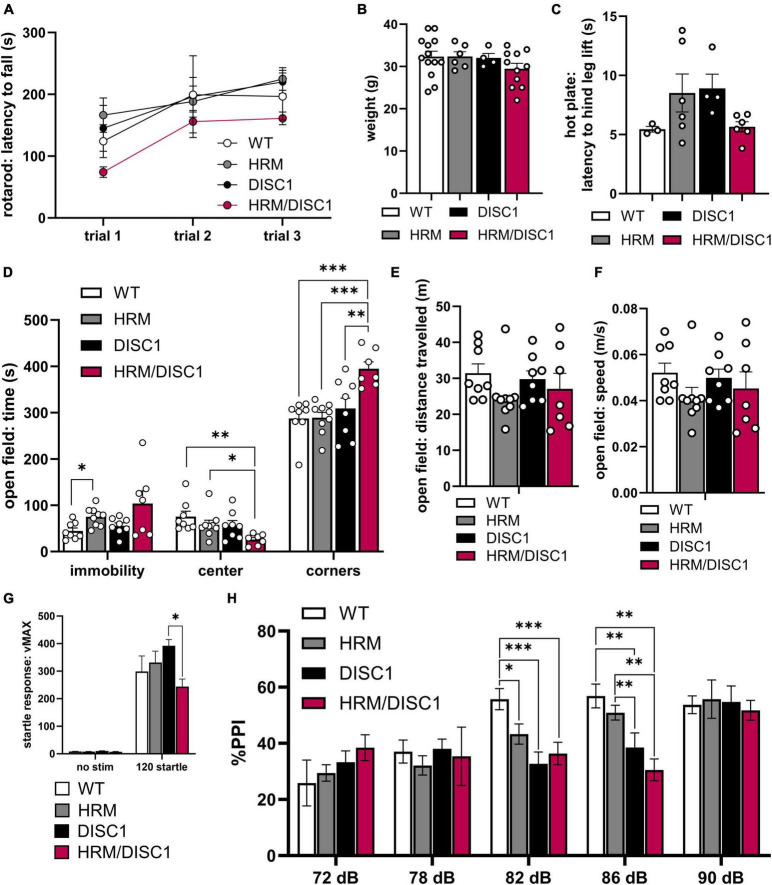
Altered activity, thigmotaxis, and prepulse inhibition in HRM/DISC1 mice. **(A)** Latency (s) to fall from rotarod with 4 m/s acceleration ramp. Trials were administered on the same day, approximately 30 m apart (*n* = 3–7). **(B)** Weight (g) of 5–6 month old mice. **(C)** Latency (s) to hind leg lift or flick on a fixed 55°C hot plate. **(D)** Open field: times (s) immobile in any part of the apparatus, time in the center, and time in the corners. **(E)** Distance (m) traveled in the open field. **(F)** Average speed (m/s) in the open field. **(G)** Startle response measured as maximum velocity (vMAX) in response to a 120 dB white noise pulse with no prepulse (*n* = 6–10). **(H)** Pre-pulse inhibition, measured as percent reduction in startle response compared to mean 120 dB vMAX when presented with a prepulse 100 ms before main 120 dB pulse (*n* = 6–10). Outliers removed by individual data points falling greater than 2 SDs from the mean. Each point represents a mouse. **(B–F)** Data represented as mean ± SEM. *p < 0.05, **p < 0.01, ***p < 0.001.

We first assessed latency to fall in a three-trial rotarod test as a measure of locomotor coordination. A repeated measures ANOVA was significant for trial [*F*(2,30) = 15.365, *p* < 0.001], but there was no interaction between trial and genotype [*F*(6,30) = 0.705, *p* = 0.648], indicating that all groups improved performance comparatively over the three trials (Figure 2A). We noted no weight differences between any groups [*F*(3,33) = 1.167, *p* = 0.339, one-way ANOVA] (Figure 2B). Nociception also appeared normal, as we found no differences in latency to hind leg lift in a hot plate test [*F*(3,18) = 2.256, *p* = 0.090, Welch’s ANOVA] (Figure 2C).

We examined immobility in the open field (OF) and found group differences using Welch’s ANOVA [*F*(3,14.449) = 4.221, *p* = 0.025] to account for significant differences in variability between groups [*F*(3,28) = 6.670, *p* = 0.002, Levene’s ANOVA] (Figure 2D). Despite a trend toward HRM/DISC1 mice showing greater immobility, this difference was not significant (*p* = 0.242, Games–Howell correction). DISC1 mice performed similarly to WT (*p* = 0.635), and HRM mice spent more time immobile (*p* = 0.023). We also compared time spent exploring the center of the OF as a measure of exploratory behavior. A one-way ANOVA revealed significant group differences [*F*(3,28) = 4.112 *p* = 0.015], driven by a reduction of time spent in the center by HRM/DISC1 mice compared to any other group (*p* = 0.002 vs. WT, *p* = 0.028 vs. HRM, *p* = 0.041 vs. DISC1, Fisher’s LSD *post-hoc*). This HRM/DISC1 thigmotaxis is a canonical sign of anxiety-like behavior in mice (Simon et al., 1994). We followed up on this by comparing time spent in the corners, finding an even more pronounced difference between HRM/DISC1 mice and WT or single mutant groups [*F*(3,28) = 8.871, *p* < 0.001, one-way ANOVA] (*p* < 0.001 vs. WT, *p* < 0.001 vs. HRM, *p* = 0.007 vs. DISC1, Fisher’s LSD *post-hoc*) (Figure 2D). We observe no changes in overall distance traveled [*F*(3,28) = 0.989, *p* = 0.412] or speed [*F*(3,28) = 0.9951, *p* = 0.409] between groups (Figures 2E, F).

We next tested pre-pulse inhibition, a measure translatable to sensory gating deficits in the clinical presentation of SCZ. There was an effect of sound level [*F*(4,60) = 9.985, *p* < 0.0001] but no interaction between decibel level and genotype [*F*(12,60) = 0.649, *p* = 0.138] in a repeated measures ANOVA. We found significant effects of genotype in pre-pulse inhibition in response to the 82 dB [*F*(3,25) = 6.664, *p* = 0.002, one-way ANOVA] and 86 dB [*F*(3,25) = 7.122, *p* = 0.001, one-way ANOVA] prepulses. Following the 82 dB prepulse, inhibition of the startle response was significantly lower in all experimental groups compared to WT (*p* = 0.031 vs. HRM, *p* < 0.001 vs. DISC1, *p* = 0.003 vs. HRM/DISC1, Fisher’s LSD *post-hoc*). Following the 86 dB prepulse, inhibition of the startle response was lower in HRM/DISC1 mice compared to WT (*p* = 0.001), and HRM (*p* = 0.003). Inhibition was also lower in DISC1 mice compared to WT (*p* = 0.005), and HRM (*p* = 0.028) (Figure 2H).

Interestingly, we also found differences in startle response to the 120 dB standalone pulse [*F*(3,10.916) = 5.183, *p* = 0.018, Welch’s ANOVA] due to a reduced startle response in HRM/DISC1 compared to DISC1 mice (*p* = 0.017, Games–Howell correction) but not compared to WT (Figure 2G).

### 2.2 HRM/DISC1 mice display normal affective behaviors but reduced preference for social novelty

**FIGURE 3:**
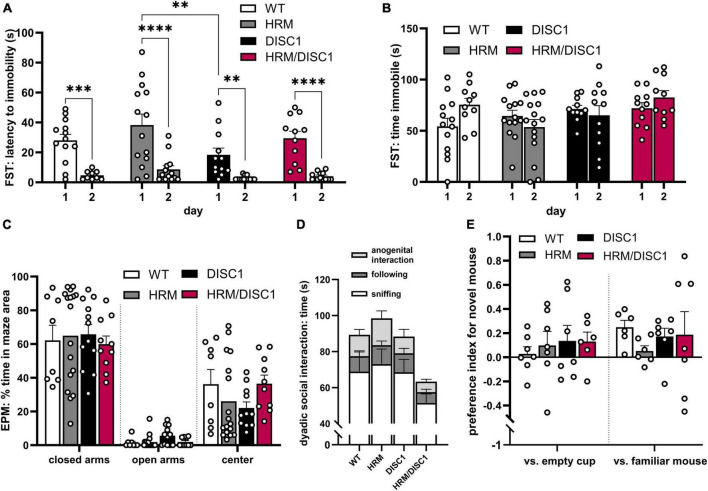
HRM/DISC1 mice display normal affective behaviors but reduced preference for social novelty. **(A)** Porsolt forced swim test was used to evaluate depression-like behavior. Latency (s) to the first instance of immobility >2 s, after the first 30 s in the chamber. **(B)** Total time (s) immobile during the last 2 min of the FST. **(C)** Elevated plus maze was used to evaluate anxiety-related behavior. Time spent in each area of the EPM, expressed as percent of the total testing time. **(D)** Social interaction in the dyadic test. Types of interactions were manually scored by a blinded researcher. Interaction types stacked to visualize total social behaviors (*n* = 7–9). **(E)** Baseline social interaction in the 3-chamber social novelty test, calculated as preference index for a stimulus mouse. The stimulus was a novel juvenile male mouse in one cup versus an empty cup, then a new novel juvenile mouse in one cup versus the previously introduced (now familiar) mouse in another cup. Preference index calculated as: [(interaction time with stimulus cup – interaction time with control cup) / total time spent interacting with either cup]. Interaction was scored by a blinded researcher for a 10-min period, following a 5-min acclimation period during which no stranger mouse was present. Outliers removed by individual data points falling greater than 2 SDs from the mean. Two mice with outliers in multiple measures were wholly removed from FST data. Data represented as mean ± SEM. **(A–C,E)** Each point represents a mouse. **p < 0.01, ***p < 0.001, ****p < 0.0001.

We next evaluated affect and sociability. To detect depression-like behavior, we used the Porsolt forced swim test, measuring latency to immobility and total time immobile. Repeated measures ANOVA with the Greenhouse–Geisser correction showed a significant reduction in the latency to immobility between day 1 and day 2 of the test [*F*(1,41) = 76.984, *p* < 0.001], but latency to immobility did not differ significantly between groups [*F*(3,41) = 1.022, *p* = 0.394] (Figure 3A). Total time immobile did not differ across days [*F*(1,41) = 0.871, *p* = 0.356] or across genotypes [*F*(3,41) = 2.272, *p* = 0.095] (Figure 3B).

To evaluate anxiety-like behavior and follow up on the marked thigmotaxis in the OF test, we measured thigmotaxis in the elevated plus maze (EPM) (Figure 3C). Using one-way ANOVAs, we found no significant differences in the time spent in the center [*F*(3,21.335) = 2.182, *p* = 0.120], closed arms [*F*(3,21.022) = 0.221, *p* = 0.88], or open arms [*F*(3,21.785) = 1.656, *p* = 0.206] of the maze.

As deficits in normal social behavior have been identified in studies of individual HRM and DISC1 mutant mouse models, we used two types of social interaction tests to evaluate social behaviors. In the dyadic test, mice paired randomly between the groups were allowed to explore the OF together, and social behaviors were monitored and recorded. While there were no significant differences in one-way ANOVA results between genotypes in anogenital interaction [*F*(3,25) = 1.299, *p* = 0.297], following [*F*(3,25) = 0.732, *p* = 0.543], sniffing [*F*(3,25) = 1.332, *p* = 0.286], or total social time [*F*(3,25) = 1.367, *p* = 0.276], there was a large overall effect size for total social interaction (Cohen’s *f* = 0.4116), indicating a trend toward lower social interaction in HRM/DISC1 mice compared to all other groups (Figure 3D).

A 3-chamber social novelty test was used to evaluate social interest in a juvenile male mouse, as well as preference for novel versus familiar mice. Baseline social preference for a stimulus mouse (a novel juvenile male) in a wire cup versus an empty cup was first established. A repeated measures ANOVA showed a significant preference for the stimulus mouse over the empty cup [*F*(1,23) = 4.614, *p* = 0.042], but no interaction with genotype [*F*(3,23) = 0.335, *p* = 0.877] (Figure 3E). During the second portion of the test a new stimulus mouse was introduced, and the now-familiar stimulus mouse placed in the opposite cup. A repeated measures ANOVA showed a significant preference for the novel stimulus mouse [*F*(1,22) = 5.231, *p* = 0.032], but no interaction with genotype [*F*(3,22) = 0.335, *p* = 0.800] (Figure 3E). However, observing the behavior of each group independently, only WT mice showed a statistically significant preference for the novel mouse [*t*(11) = 4.298, *p* = 0.001, independent samples *t*-test]. HRM [*t*(11) = −0.560, *p* = 0.586], DISC1 [*t*(12) = 1.532, *p* = 0.152], and HRM/DISC1 [*t*(12) = 0.611, *p* = 0.553] groups spent roughly the same amount of time interacting with the familiar and novel stimulus mice.

### 2.3 Cognitive behavioral assessment of HRM/DISC1 mice

The cognitive symptoms of SCZ are highly indicative of patient outcome, and determining what level of cognitive impairment, if any, is present in our model is essential for determining validity. To this end, we used several measures to detect differences in learning and memory in WT, HRM, DISC1, and HRM/DISC1 mice.

**FIGURE 4:**
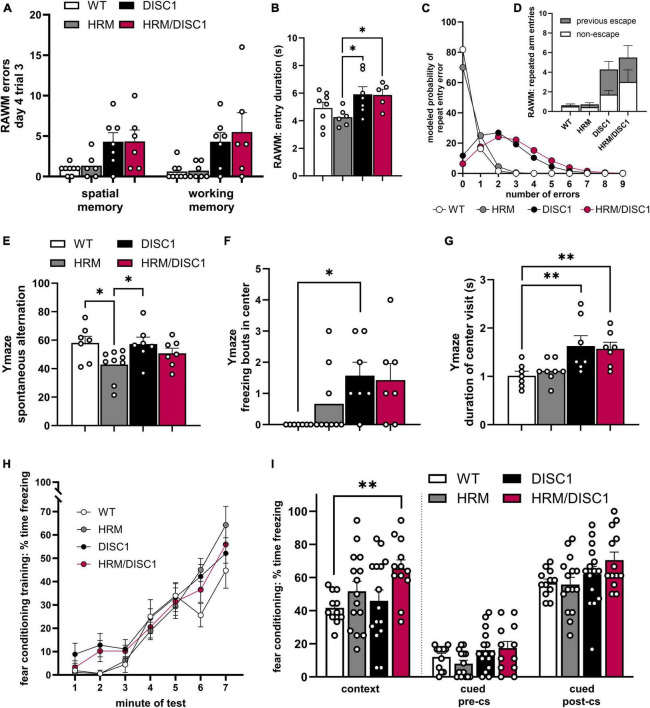
Cognitive behavioral assessment of HRM/DISC1 mice. Radial arm water maze: quantification of **(A)** spatial memory errors, classified as any entry into an arm of the maze that never contained an escape platform; working memory errors, classified as repeated entry into any arm, and **(B)** escape latency. **(C)** Poisson regression model predicting the number of repeated arm entry errors for each group, and **(D)** visualization of actual entry arm errors showing the proportion of re-entry into the most recent escape arm. Y-maze: **(E)** quantification of € spontaneous alternation, calculated by (alternating triads / total possible triads) × 100, **(F)** freezing bouts of at least 2 s in the center area of the maze, and **(G)** average duration (s) of visits to the center of the maze. Classical fear conditioning: quantification of percent time freezing during **(H)** training, **(I)** the contextual test, and **(J)** the cued test before and after presentation of the conditioned stimulus in a novel context. Data represented as mean ± SEM. **(A,B,E–G,I)** Each point represents a mouse. *p < 0.05, **p < 0.01.

In the radial arm water maze (RAWM), we examined the number of errors made during the last trial on the last day of testing. During this trial, only one of three possible escape platforms remains, the previous two platforms having been removed upon successful navigation of the test mouse to the correct arm. While a clear trend in performance emerged, the individual performance of DISC1 and HRM/DISC1 mice was more variable compared to WT and HRM groups. We counted the number of entries into arms that never contained a platform as spatial memory errors, and while there were significant group differences [*F*(3,10.531) = 4.320, *p* = 0.032, Welch’s ANOVA], Games–Howell *post-hoc* tests were not significant for individual differences between genotypes (Figure 4A). We did find significant group differences in working memory errors (defined as repeated entries in the same arm of the maze) [*F*(3,11.546) = 4.320, *p* = 0.037, Welch’s ANOVA], with both DISC1 and HRM/DISC1 mice showing significant deficits compared to WT mice (*p* < 0.001) (Figure 4B).

While the RAWM results suggest a potential working memory deficit, in the Y-maze we found no alteration in spontaneous alternation between HRM/DISC1 and any other group. We detected a significant group difference [*F*(3,29) = 3.113, *p* = 0.043] driven by a reduction in spontaneous alternation in the HRM group compared to WT (*p* = 0.014) and DISC1 (*p* = 0.018) (Figure 4E).

To address the incongruity between our working memory results and further investigate executive dysfunction in our model, we also asked if the errors we classified as working memory errors in the RAWM might be evidence of perseveration. To begin to address this question, we revisited our RAWM results and calculated an estimated time spent per arm entry, finding significant group differences in entry duration [*F*(3,22) = 3.053, *p* = 0.050], reflecting a lower entry duration in the HRM group compared to DISC1 (*p* = 0.015) and HRM/DISC1 (*p* = 0.028) (Figure 4C). We also investigated the nature of the repeated arm entries and found that while most WT mice made zero repeat arm entry errors during the last trial, HRM/DISC1 made 33 in total, 18 (54.55%) of which were repeated entries into the arm they most recently used to escape (Figure 4D). Due to the many zero counts for repeated arm entries, we modeled a Poisson regression, and determined via Wald Chi-square that genotype is a significant predictor of repeat arm entries [*W*(3) = 34.113, *p* < 0.001]. We found that, compared to WT, the predicted repeat entry rate for HRM/DISC1 [*W*(1) = 18.892, *p* = <0.001, *B* = 2.621, SEB = 0.603] and for DISC1 [*W*(1) = 15.339, *p* = <0.001, *B* = 2.372, SEB = 0.606] were significantly greater, while the HRM genotype was not a significant factor [*W*(1) = 0.63, *p* = 0.427]. HRM/DISC1 mice are predicted to have a repeat entry error rate 13.75 times higher than WT. This supports the idea that further investigation of perseveration or a more extensive phenotype of executive dysfunction is merited.

To further address the ambiguity in behavioral results, we investigated decision-making behavior in the Y-maze by counting the number of freezing bouts in the center of the maze, as well as the average duration of visits to the center. We used an independent-samples Kruskal–Wallis test to determine differences, finding a significant difference in distribution [χ^2^(3) = 10.640, *p* = 0.014]. *Post-hoc* testing with the Dunn–Bonferroni correction showed that, compared to WT, this difference is driven by the increase in DISC1 group (*z* = −12.571, *p* = 0.021), and a trend toward an increase in the HRM/DISC1 group (*z* = −10.786, *p* = 0.074) (Figure 4F). As expected, this increase in freezing was reflected in an increased length of time per visit to the center zone [*F*(3,29) = 3.152, *p* = 0.042, one-way ANOVA] driven by differences between WT and DISC1 (*p* = 0.013) and WT and HRM/DISC1 groups (*p* = 0.023) (Figure 4G).

We used classical fear conditioning to investigate associative learning and memory in our model. During training, a repeated measures ANOVA with the Greenhouse–Geisser correction was significant by minute of the test for freezing [*F*(3.756) = 82.915, *p* < 0.000] reflecting the expected increase in freezing following presentation of the tone-shock pairing, but there was no significant interaction between time and genotype [*F*(11.267) = 1.543, *p* = 0.117], indicating intact learning (Figure 4H). In the contextual fear conditioning test, we found increased freezing in the HRM/DISC1 group compared to WT, confirmed by a significant Welch’s ANOVA [*F*(3,26.363) = 5.440, *p* = 0.005] with the Games–Howell *post-hoc* (*p* = 0.004) (Figure 4I). In the cued test, there was a non-significant trend toward increased freezing in HRM/DISC1 mice both during the pre-CS period [*F*(3,49) = 2.126, *p* = 0.109] and cue presentation [*F*(3,51) = 2.323, *p* = 0.086] (Figure 4I). Given the presence of increased freezing in the OF, baseline changes in anxiety may contribute to these results. While we found no differences in basic measures of locomotion in the OF, it is possible that these mice may have more subtle changes in meandering or angular velocity that might inform interpretation of these results and should be further explored.

### 2.4 HRM/DISC1 mice display altered GABA_*A*_ receptor expression and a paradoxical reaction to diazepam

**FIGURE 5:**
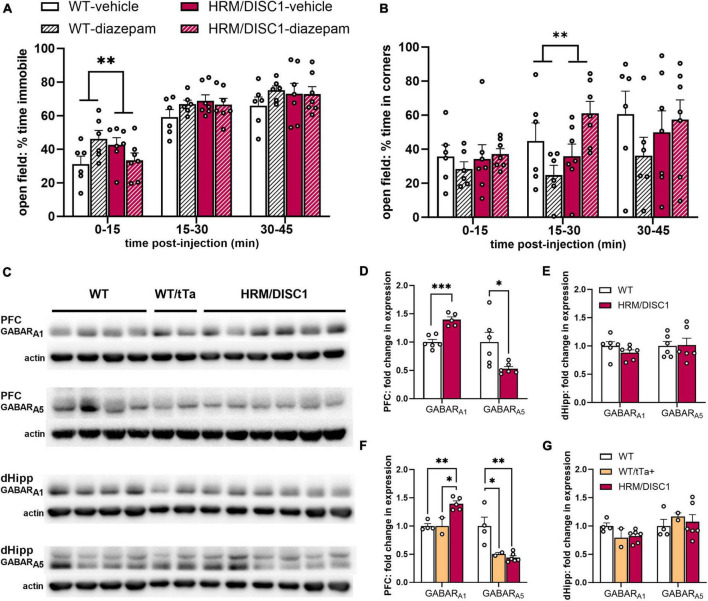
HRM/DISC1 have a paradoxical reaction to diazepam treatment and reduced expression of subunit A1 containing GABA receptors. Following subcutaneous diazepam injection, **(A)** immobility and **(B)** time (s) spent in the corners of the open field over 45 m, split into 15 m bins. **(C)** Western blots for GABA receptor subtypes A1 and A5 in the prefrontal cortex (PFC) and the dorsal hippocampus (dHipp) of WT, WT/tTa+, and HRM/DISC1 mice. **(D,E)** Quantification of GABA receptor subtypes A1 and A5 in the PFC and dHipp. Bands were normalized to actin and are expressed as fold change from control. The WT control group includes WT and WT/tTa mice. **(F,G)** Quantification of GABA receptor subtypes A1 and A5 in the PFC and dHipp, with WT and WT/tTa mice shown as separate groups. Data expressed as fold change from WT average. Outliers removed by individual data points falling greater than 2 SDs from the mean. Bars indicate mean ± SEM. Each point represents a mouse. *p < 0.05, **p < 0.01, ***p < 0.001.

To determine whether the reduction in activity across multiple behavioral tests could be attributed to anxiety, we treated separate cohorts of WT and HRM/DISC1 mice with the classic anxiolytic diazepam. We administered a single 1.5 mg/kg dose via subcutaneous injection and allowed mice to explore the OF. We recorded behavior for 45 min to account for difference in subcutaneous absorbance of diazepam. Surprisingly, we found that the diazepam treatment exerted opposing effects on WT and HRM/DISC1 mice. A two-way ANOVA showed a significant interaction between diazepam treatment and genotype during the first 15 min of testing [*F*(1,20) = 9.156, *p* = 0.007], as treatment caused an increase in immobility for WT mice and decrease for the HRM/DISC1 group, compared to vehicle-only treatment (Figure 5A). Treatment also had opposing effects on the time spent in the corners of the maze from 15 to 30 min into testing [*F*(1,20) = 8.160, *p* = 0.010], and we observed a trend in this behavior during the first 15 min of testing [*F*(1,20) = 3.986, *p* = 0.06] (Figure 5B). As expected, WT mice spent less time in the corners during the first 30 min of the test with diazepam treatment, while HRM/DISC1 mice spent more time in the corners.

As diazepam acts directly on GABA_*A*_ receptors (Costa and Guidotti, 1996), we followed up on this finding by investigating GABA_*A*_ receptor subtype expression in the hippocampus and PFC of WT and HRM/DISC1 mice using Western blotting (Figure 5C). We focused on GABA receptor A1 (GABAR_*A*1_) and A5 (GABAR_*A*5_), as Guidotti’s group demonstrated a paradoxical increase in locomotion with diazepam treatment in socially isolated mice, and found associated changes in the expression of these receptors (Pinna et al., 2006). In the PFC, we found increased expression of GABAR_*A*1_ [*t*(9) = 5.818, *p* < 0.001], and decreased expression of GABAR_*A*5_ [*t*(5.492) = 2.654, *p* = 0.041] (Figure 5D). We found no changes in the hippocampus in GABAR_*A*1_ [*t*(10) = 0.775, *p* = 0.217] or GABAR_*A*5_ [*t*(10) = 0.118, *p* = 0.909] (Figure 5E).

Because we included WT/tTa animals in our control group, we also reanalyzed our Western blot results to determine whether the expression of tTa had any effects on GABA receptor subtype expression, though interpretation of these results is limited by the low number of animals in the split WT and WT/tTa+ groups. With WT and WT/tTa groups considered separately, the GABAR_*A*1_ expression change we saw in the PFC persisted overall [*F*(2,10) = 15.05, *p* = 0.002], with both WT (*p* = 0.003) and WT/tTa (*p* = 0.01) expression significantly lower than the HRM/DISC1 group, and nearly identical to each other (*p* = 0.999) (Figure 5F). However, while PFC GABAR_*A*5_ expression also remained different between all three groups [*F*(2,11) = 10.81, *p* = 0.004], compared to WT, expression was reduced in both the WT/tTa (*p* = 0.036) and HRM/DISC1 (*p* = 0.004) groups, while in this case the WT/tTa and HRM/DISC1 groups were nearly identical (*p* = 0.917) (Figure 5F). This suggests the possibility of a confounding effect of tTa expression that would need to be confirmed with further testing on a greater number of animals. We repeated these analyses on the blot data from the dorsal hippocampus, but found no differences in GABAR_*A*1_ [*F*(2,11) = 2.772, *p* = 0.115] or GABAR_*A*5_ [*F*(2,11) = 0.262, *p* = 0.775] (Figure 5G).

### 2.5 Decreased density of parvalbumin positive cells, and increased number of GAD67+/PV− cells in the medial prefrontal cortex of HRM/DISC1 mice

**FIGURE 6:**
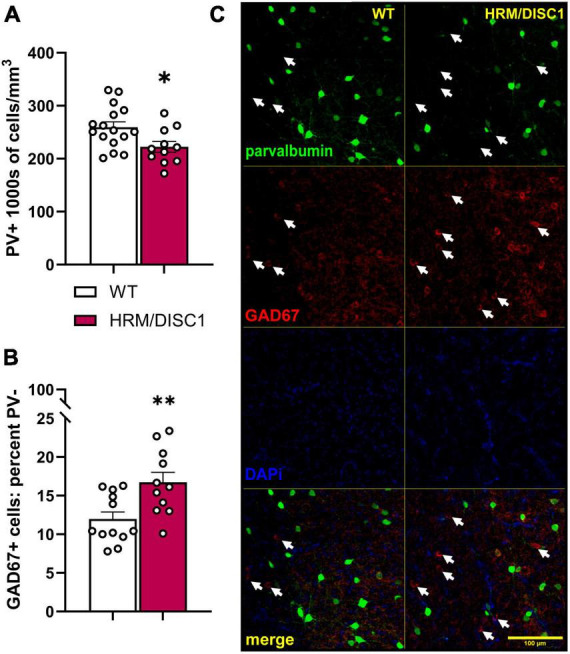
Decreased density of parvalbumin positive (PV+) cells, and increased number of GAD67+/PV– cells in the medial prefrontal cortex of HRM/DISC1 mice. **(A)** Quantification of the total number of PV+ cells, presented as 1,000 s of cells/mm^3^. **(B)** Percent GAD67+/PV– cells, calculated as number of GAD67+/PV– cells/total number of PV+ and GAD67+ cells. **(C)** Representative 20× micrographs. Arrows indicated GAD67+/PV– cells. Cells were counted exhaustively in six 40 μm sections from three WT and two HRM/DISC1 mice, using tiled, merged z-stacks of six confocal planes taken at a 2 μm interval. Each data point represents one section. Bars indicate mean ± SEM. *p < 0.05, **p < 0.01.

There is extensive evidence for alterations in inhibitory signaling in the PFC in SCZ (Selemon, 2001; Lewis et al., 2005). We investigated the effects of the combined genetic disruption on inhibitory cells in the medial PFC by determining the density of parvalbumin (PV) positive cells, and the occurrence of GAD67+/PV− cells (Figure 6C). We found a decrease in the PV+ cell density [*t*(24) = 2.549, *p* = 0.018] (Figure 6A) and an increase in the percentage of GAD67+ cells that did not express PV [*t*(21) = 3.027, *p* = 0.006] (Figure 6B).

### 2.6 Impaired maturation of adult-born neurons in the dentate gyrus of HRM/DISC1 mice

**FIGURE 7:**
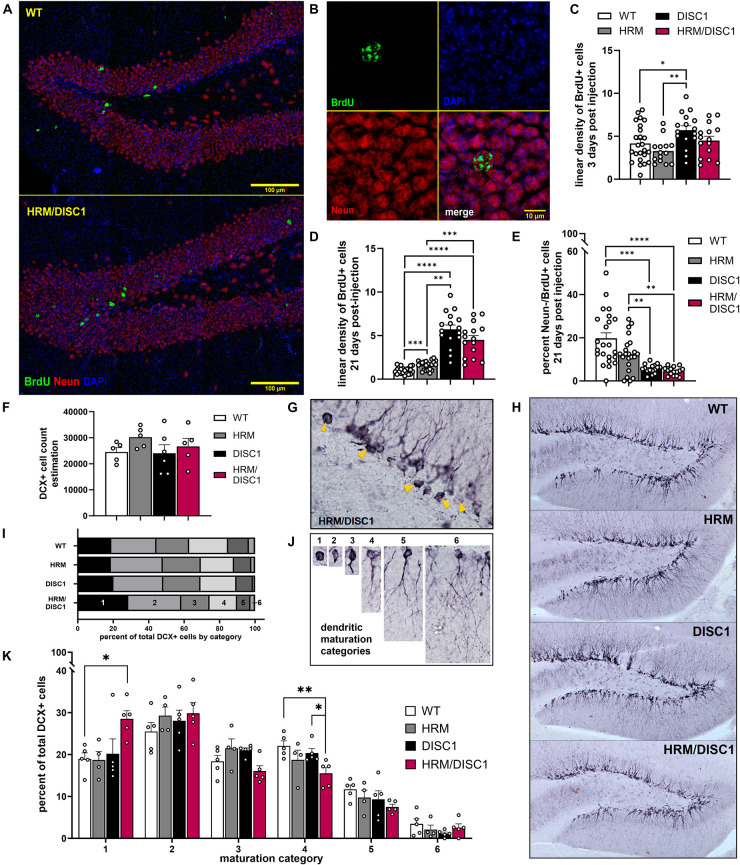
Characterization of adult neurogenesis and neuronal maturation in the HRM/DISC1 dentate gyrus. **(A)** Representative merged 40× z-stack confocal micrographs, showing NeuN (red) and BrdU+ (green) cells in the adult dentate gyrus of WT and HRM/DISC1 mice, 21 days after BrdU injection. **(B)** Representative 63× confocal micrograph showing a BrdU+/NeuN+ cell in an HRM/DISC1 brain. **(C)** Quantification of BrdU+ cells, counted in the whole hippocampus, 3 days after injection. Cells were counted exhaustively and bilaterally in four serial sections from three WT, two HRM, two DISC1, and two HRM/DISC1 brains. **(D)** Quantification of BrdU+ cells in the medial dentate gyrus of the dorsal hippocampus, 21 days after injection. Cells were counted in the granular cell layer and hilus along an approximately 1 mm length of subgranular zone, bilaterally in four serial sections from three WT, three HRM, two DISC1, and three HRM/DISC1 brains. Cells were counted from six tiled 40× confocal z-stacks with seven planes at a 2 μm interval. Linear density was calculated by dividing the number of BrdU+ cells by the measured length of the subgranular zone in each section and expressed as fold change from control. **(E)** Quantification of BrdU+/NeuN– cells, indicating adult-born cells that differentiated into non-neuronal cell types. All BrdU+ cells were assessed, and cellular colocalization was confirmed using orthographic views as necessary. **(F)** Stereological estimation of the total number of DCX+ cells in the dentate gyrus. **(G)** Representative 40× micrograph of DCX+ cells in an HRM/DISC1 brain. Arrowheads indicated proliferative, category 1 cells. **(H)** Representative 10× micrographs of DCX staining in the dentate gyrus, demonstrating the lack of dendritic outgrowth into the granule cell layer in the HRM/DISC1 hippocampus. **(I)** Proportion of DCX+ cells in each maturation category, out of total cells counted. **(J)** Representative images of cells which were categorized as: 1 – proliferative, no dendritic processes; 2 – proliferative, one short process; 3 – intermediate, medium process; 4 – intermediate, medium process that extends to the molecular layer; 5 – mature, one dendrite branching into the molecular layer; 6 – mature, delicate dendritic tree in granule cell layer. **(K)** Quantification of DCX+ cell categorization. Each data point represents one mouse. Outliers falling more than 2 SDs from the mean removed. Bars indicate mean ± SEM. *p < 0.05, **p < 0.01, ***p < 0.001, ****p < 0.0001.

Both reelin (Teixeira et al., 2012; D’Arcangelo, 2014) and DISC1 (Brandon and Sawa, 2011) are involved in neuronal migration and maturation, so we next examined adult neurogenesis, neuronal survival, and neuronal maturation in the dentate gyrus (DG) of the hippocampus. We first used the synthetic thymidine analog 5-bromo-2′-deoxyuridine (BrdU) to label dividing cells and identify changes in neurogenesis in the adult DG. We injected BrdU for 3 consecutive days and collected tissue 3 days after the last injection, then performed immunofluorescent staining to visualize newborn cells with incorporated BrdU (Figures 7A, B). We found group differences in the linear density of BrdU cells [*F*(3,68) = 4.068, *p* = 0.010, one-way ANOVA]. DISC1 mice had significantly more BrdU+ cells compared to WT (*p* = 0.017) and HRM (*p* = 0.001), while differences between HRM/DISC1 mice and both HRM (*p* = 0.097) and DISC1 (*p* = 0.090) were not significant (Figure 7C).

To evaluate survival and differentiation of adult-born cells in the DG, we performed a new set of BrdU injections, waiting 21 days to allow cells to differentiate and mature before taking tissue. We performed immunofluorescent staining for BrdU and the neuronal marker NeuN to identify mature, adult-born neurons (Figures 7D, E). We counted the total number of BrdU+ cells in the DG of the dorsal hippocampus, and the number of BrdU+ cells that did not express NeuN, indicating differentiation into non-neuronal cell types. Compared to WT, we found a large increase in the number of BrdU+ cells in HRM/DISC1 mice [*t*(40) = 4.2425, *p* = 0.0001], and a smaller proportion of BrdU+/NeuN− cells [*t*(41) = 31.742, *p* = 0.039].

We used doublecortin (DCX) immunohistochemistry to identify immature neurons, and unbiased stereological estimation to determine the total number of DCX+ cells in the DG, finding no differences [*F*(3,19) = 0.871, *p* = 0.476, one-way ANOVA] (Figure 7F). As DCX+ cells from HRM/DISC1 brains appeared to have less advanced dendritic outgrowth than other groups (Figures 7G, H), we used the schema developed by Plümpe et al. (2006) to categorize the maturation of each DCX+ cell that was counted based on dendritic outgrowth (Figures 7I, J).

We found group differences in the number of immature, proliferative DCX+ cells (Figure 7K). A repeated measures ANOVA with the Greenhouse–Geisser correction showed a significant effect interaction between category and genotype [*F*(8.798,43.992) = 2.416, *p* = 0.026]. Individual ANOVAs for the different categories revealed differences in the number of cells in categories 1 [*F*(3,18) = 3.812, *p* = 0.033] and 4 [*F*(3,18) = 3.831, *p* = 0.032], due to a large increase in the number of immature cells in the HRM/DISC1 group (*p* = 0.012 vs. WT, *p* = 0.014 vs. HRM, *p* = 0.024 vs. DISC1). This was balanced by a reduction in the number of cells in the intermediate category 4 compared to WT (*p* = 0.005) and DISC1 (*p* = 0.029), but not HRM (*p* = 0.152) (Figure 7I). There was also a trend toward differences in category 3 cells, and while it failed to reach significance [*F*(3,18) = 3.141, *p* = 0.057], the group effect size (Cohen’s *f* = 0.7262) suggests that the lower number of category 3 cells in HRM/DISC1 hippocampi may be of interest.

### 2.7 Reduced dendritic spine density in HRM/DISC1 hippocampal dentate granule cells

**FIGURE 8:**
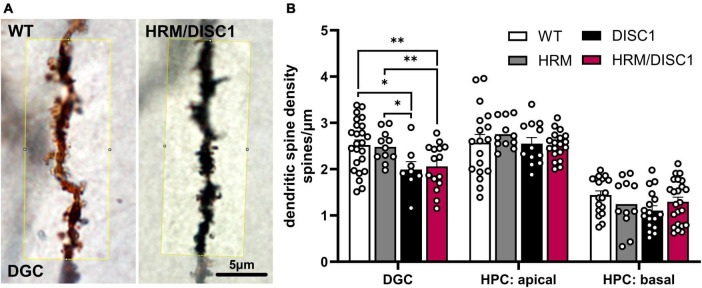
Reduced dendritic spine density in HRM/DISC1 hippocampal dentate granule cells. **(A)** Representative 100× micrographs, cropped from single planes of z-stack images of hippocampal dentate granule cells (DGC) in WT and HRM/DISC1 brains, visualized using the Golgi-Cox impregnation and staining method. Yellow box denotes ∼20 μm long counting area. Counts were normalized by the actual measured length of the segment counted to generate a linear density of spines (spines/μm). **(B)** Quantification of linear spine density in DGC, hippocampal pyramidal cell (HPC) apical dendrites, and HPC basal dendrites. Three to five brains per group were stained and imaged. Each data point represents one segment. Outliers falling more than 2 SDs from the mean removed. Bars indicate mean ± SEM. *p < 0.05, **p < 0.01.

To further investigate the effects of reelin and DISC1 disruption on dendritic spines, we used Golgi-Cox staining to visualize a subset of neurons and counted dendritic spines in dentate granule cells (DGC) and hippocampal pyramidal cells (HPC) (Figure 8A). We found no group differences in linear spine density in secondary apical or primary basal dendrites of HPCs. While we noted no group differences in a one-way ANOVA between all experimental groups, we noted that an individual independent samples *t*-test reflects a reduction in DISC1 basal HPC linear spine density compared to only to WT [*t*(39) = 2.132, *p* = 0.032]. We found group differences in spine density in DGC [*F*(3,57) = 4.261, *p* = 0.009], driven by a reduction in DISC1 (*p* = 0.011) and HRM/DISC1 (*p* = 0.007) brains compared to WT, and DISC1 (*p* = 0.036) and HRM/DISC1 (*p* = 0.036) brains compared to HRM (Figure 8B).

## 3 Discussion

In this study, we consider the complex genetic landscape of SCZ. We increased the genetic load of putative SCZ vulnerability genes by combining expression of dominant-negative C-terminal truncated human DISC1 with reelin haploinsufficiency.

Working memory deficits are common in SCZ patients, and are associated with dorsolateral PFC dysfunction (Goldman-Rakic, 1999; Harvey et al., 2003). While our molecular results suggest that HRM/DISC1 mice have some inhibitory imbalance in the PFC, we did not find a robust working memory deficit. HRM/DISC1 and DISC1 mice made more repeat-entry type errors in the RAWM but showed normal spontaneous alternation in the Y-maze. While repeated arm entries in the RAWM could be attributed to a working memory deficit, we observed that many errors were repeated entries into the most recent escape arm which could also indicate intact working memory performance confounded by perseveration.

Abnormal gamma-band oscillations (McNally and McCarley, 2016) is a consistent finding in SCZ patients. Gamma oscillations (∼25–100 hz) are generated and synchronized through inhibitory feedback by fast-spiking PV+ interneurons onto pyramidal neurons during various cognitive processes (Tamás et al., 2000; Sohal et al., 2009; Nakamura et al., 2015), and are involved in sensorimotor integration (Gandal et al., 2012). Reduction of GAD67 in PV+ cells of the dorsolateral PFC is common in SCZ patients, and deficient inhibitory output in the PFC may impact working memory and other cognitive functions. Human fMRI studies comparing PFC activation during working memory task performance between controls and SCZ patients have demonstrated PFC inefficiency—some SCZ patients perform similarly to controls but use greater PFC resources (Callicott et al., 2003). Our model expresses c-terminal truncated DISC1 in the PFC (Schurov et al., 2004; Holley et al., 2013), and reelin, expressed by GABAergic interneurons, is reduced by ∼50% in the HRM. We found a reduction in the total number of PV+ cells in the medial PFC of HRM/DISC1 mice, and an increased proportion of GAD67+/PV− cells, suggesting that the alteration in cell number is specific to PV+ cells, rather than an overall decrease in GABAergic cells. As such, reduced inhibitory output by PV+ GABAergic interneurons onto PFC pyramidal neurons is a potential mechanism for altered behavior in the HRM/DISC1 model.

Pre-pulse Inhibition (PPI) of the acoustic startle response is used as a translatable measure of sensorimotor gating in both human and animal testing. We found a reduction in startle response in HRM/DISC1 mice, along with decreased PPI in all groups at the 82 db prepulse level, and in DISC1 and HRM/DISC1 groups at the 86 dB prepulse level. Abnormal startle response and PPI has been reported in the HRM (Tueting et al., 1999), but has not been consistently reproduced, and is sensitive to age and PPI modality (Barr et al., 2008). While PPI deficits have been reported in several models of DISC1 disruption, they are highly variable (Hikida et al., 2007; Pletnikov et al., 2008).

Reduced exploratory behavior in the OF and decreased mobility in other tests complicate interpretation of behavioral phenotypes. This is particularly relevant in classical fear conditioning, as freezing behavior is used as a measure of fear to indicate memory of the aversive stimuli. Potential fear conditioning deficits could be masked by an overall increase in freezing behavior. Future work using this and related models should include an expanded range of cognitive tests that are sensitive to perseveration, attention, cognitive flexibility, and decision-making using outcome measures that do not rely on freezing.

We considered the possibility that the reduction in exploratory behavior and immobility observed in HRM/DISC1 mice might be related to anxiety, despite normal EPM performance, as a floor effect could mask anxiety. Administration of diazepam unexpectedly failed to rescue the exploratory behavior deficit in HRM/DISC1 mice, increasing time in the corners of the apparatus and increasing mobility. The increase in immobility in WT mice could possibly be attributed to some level of sedation. We expected that the observed increase in the expression of A1-containing GABA receptors in HRM/DISC1 mice might further increase this effect, yet we observed the opposite. While these results point to changes in inhibitory signaling in HRM/DISC1 mice, it is limited to the expression of only A1 and A5 GABA receptor subtypes, and a complete picture of overall GABA receptor expression is necessary. The combination of WT and WT/tTa animals in our control group also represents a potential limitation of this study, as demonstrated by the potential difference in PFC GABA_*A*5_ expression between WT and WT/tTa. However, only 2 WT/tTa samples were analyzed, and further exploration will be necessary to determine the full impact, if any, of tTa on these measures.

Defects in adult hippocampal neurogenesis have been reported in SCZ patients and in multiple animal models. While we found no overall decrease in the number of proliferating cells in the adult hippocampus of HRM/DISC1 mice, we found that immature neurons have reduced dendritic outgrowth during the DCX-expressing stage of maturation. We also found an increased number of BrdU+ cells in the DG of the dorsal hippocampus of the HRM/DISC1 brain, a lower proportion of which were NeuN−, indicating differentiation into non-neuronal cell types. It is also possible that some cells of neuronal origin fail to survive in this model. Taken together, the processes underlying the survival and maturation of adult-born neurons in the hippocampus appear altered in the HRM/DISC1 brain. Programmed cell death (PCD) is an essential regulatory function involved in adult neurogenesis, with the most extensive PCD occurring during synaptogenesis in postmitotic neurons, resulting in a loss of 30%–70% of immature neurons (Brandt et al., 2003; Ryu et al., 2016).

We found reduced spine density in DGC of both DISC1 and HRM/DISC1 mice, and no differences in spine density in basal or apical dendrites of HPCs. Reduced basal HPC spine density has been reported in the HRM (Liu et al., 2001), and reduced spine density has been observed in many DISC1 mutant models (Tropea et al., 2018). Interestingly, Pletnikov’s group reports an increase in spine density in DGCs in the c-terminal truncated DISC1 model (Ayhan et al., 2011), and reduction in response to maternal immune activation in a separate study (Abazyan et al., 2010).

One possible reason for the incongruity between dendritic spine counts in the c-terminal truncated DISC1 model is reactivity to an environmental factor like stress or social isolation. Additionally, physical exercise is well-known to increase dendritic spine density in the hippocampus (Stranahan et al., 2007). As HRM have been described as having a dampened responsiveness to environmental challenges (Laviola et al., 2009), one possibility for our results is that voluntary exercise on wheels present in cages during these experiments increased spine density in all groups, while DISC1 and HRM/DISC1 mice may be more sensitive to social isolation or stress compared to HRM.

We found only subtle changes in sociability. While this is contrary to results of altered social behavior in the dyadic test reported for the individual DISC1 mouse (Pletnikov et al., 2008), this can potentially be attributed to differences in methodology. Our dyadic social interaction tests paired non-littermate mice randomly from all groups, and while we measured social behaviors initiated by each mouse, these behaviors were likely limited by variations in social reciprocation and the mobility of mice. Our 3-chamber sociability test eliminated some of these issues but measured only male to juvenile male sociability. We found no difference in baseline social interaction in this test but found a reduced preference for social novelty. Only the WT group preferred the novel mouse, while HRM, DISC1, and HRM/DISC1 mice spent roughly the same amount of time with the familiar and novel mice.

Schizophrenia has a temporal profile of susceptibility, with behavioral changes beginning in the pubertal period (Woods, 1998). Determining which of these behavioral, cellular, and molecular changes we show in HRM/DISC1 mice are present in juvenile mice will be useful for determining how they contribute to susceptibility to the environmental insults. This study is also limited to male mice. Sex differences in HRM/DISC1 mice are likely present, and have been reported in both individual HRM and DISC1 models, including cognitive deficits (Ayhan et al., 2011) and depression (Pletnikov et al., 2008) exclusive to female DISC1 mutant mice.

In summary, we identified alterations in neuronal maturation and dendritic outgrowth in the hippocampus, alterations in GABAergic cells in the PFC, and a complex behavioral profile best described by intact affect, a strong sensorimotor gating deficit, and subtle changes in cognition in the HRM/DISC1 mouse. This study provides valuable insight into the contributions of reelin and DISC1 to adult neurogenesis and behavior.

## 4 Materials and methods

### 4.1 Animals

To generate a gene–gene interaction model of DISC1 and reelin disruption, we bred double transgenic mice with inducible mutant human DISC1 with heterozygous reeler mice (HRM) to produce a triple transgenic mouse line.

The inducible human DISC1 mouse was provided by M.V. Pletnikov’s group (Johns Hopkins University School of Medicine, Baltimore, MD, USA). In brief, a Tet-off double transgenic system was used, and founder mutant human DISC1 mice were bred with single transgenic B6;CBA-Tg(Camk2a-tTA)1Mmay/j mice (Jackson Laboratory, RRID:SCR_004633) expressing tetracycline-controlled transactivator driven by the calcium-calmodulin-dependent kinase II-α (CAMKIIα) promoter. This results in constitutive expression of mutant human DISC1 primarily in excitatory forebrain neurons that may be turned off my administration of doxycycline (DOX) in mouse chow (see Pletnikov et al., 2008 for detailed methods; Mayford et al., 1996; Pletnikov et al., 2008). DOX was not administered for these experiments. Expression of mutant human DISC1 and tTa were confirmed via polymerase chain reaction. “DISC1” refers to mutant human DISC1 and tTa positive mice throughout the manuscript.

HRM (B6C3Fe a/a-Relnrl/+ strain) mice were originally obtained from Jackson Laboratory. DISC1 mice were crossed with HRM to obtain a double transgenic mouse line and were back-crossed to C57Bl/6J wild type mice.

In order to minimize the number of mice necessary, we included mice expressing only tTa and not DISC1, or only DISC1 and not tTa in the WT and HRM control groups. We could detect no behavioral differences within these groups, however, we found potential differences between WT and WT/tTa groups in our GABA receptor subtype assay. These results reflect only two tTa mice but will be an important consideration moving forward.

All animal testing procedures and care followed the NIH guidelines and were approved by the University of South Florida’s Institutional Animal Care and Use Committee.

### 4.2 Behavioral testing

All behavioral tests were performed on groups of male mice, 2–6 months of age. Mice were group housed in standard LD, fed standard mouse chow *ad libitum*, and all tests were performed during the animals’ light cycle. Mice were allowed 30–60 min to acclimate to behavioral testing areas before beginning tests. Each apparatus was thoroughly cleaned with 70% ethanol between mice to minimize scent cues. White noise was played in behavioral areas.

#### 4.2.1 Open field

Exploratory behavior and mobility were tested in the OF. Mice were placed in the center of a (27 × 27 cm) box evenly lit from above and allowed to explore for 10 min. Behavior was recorded and tracking was scored automatically by ANY-maze software (RRID:SCR_014289).

#### 4.2.2 Hot plate

Nociception was evaluated using a hot plate test (Ugo Basile), to ensure that allodynia or hyperalgesia effects would not impact fear conditioning results that were dependent on foot shock. Plate temperature was fixed at 55°C. Mice were carefully monitored for hind-leg flick response (or any other indicator of distress) and removed promptly.

#### 4.2.3 Pre-pulse inhibition

Pre-pulse inhibition testing was performed to assess sensory gating. Animals were placed in a clear, cylindrical restrainer (Panlab, Barcelona, Spain) inside a sound-attenuation chamber. Animals were acclimated for 5 min in the presence of 65 dB white noise then were presented with seven trial types in a pseudorandom order with an inter-trial interval of 10–20 s: a 40 ms, 120 dB sound burst (startle); 74, 78, 82, 86, and 90 dB acoustic pre-pulses preceding a 120 dB sound burst by 100 ms; and no stimulus. Startle responses were measured as force and latency to response using an accelerometer located beneath the restraint platform. Percent PPI was calculated using the formula: [100 − (response to pre-pulse + 120 dB) / (response for 120 dB alone) × 100].

#### 4.2.4 Forced swim test

Forced swim test (FST) was used to measure depression-like behavior. A 2-day testing paradigm was used, in which animals learned on the first day that escape is not possible. Each day, animals were placed in warm water in a 5 L beaker (27 ± 2°C, diameter: 20 cm, water depth: 12 cm), and allowed to remain for 6 min. The beaker was placed within the OF apparatus to prevent visualization of the researcher or other animals during testing. The test was recorded by overhead digital camera, then immobility was scored by a researcher blinded to the experimental conditions. Latency to immobility was quantified following a 30 s period of acclimatization, and percent time spent immobile was quantified during the last 2 min of the test.

#### 4.2.5 Elevated plus maze

Elevated plus maze (EPM) was used to evaluate anxiety. The apparatus consisted of four arms, elevated 40 cm from the surface: two opposite open, well-lit arms (30 × 5 cm), and two enclosed arms (30 × 5 × 15 cm), each attached to an open (4.5 cm square) center platform. Animals were placed on the center platform and allowed to explore for 5 min. Activity was monitored by an overhead digital camera, and ANY-Maze software was used to record and analyze mice behavior. Anxiety levels were assessed by ratio of time spent in the open:closed arms.

#### 4.2.6 Social interaction

Social behavior was assessed using social interaction in an OF apparatus. Sex-matched non-cagemates were placed in a lit box (40 × 40 × 27 cm) for 10 min. Activity was recorded using a digital camera, and the video evaluated by a researcher blinded to the experimental conditions. For each animal, time spent sniffing, following, and engaging in anogenital interaction was recorded, and a total social time calculated as the sum of these activities. The animal being assessed was considered to be sniffing if its head was within 2 inches of the other animal, following if moving in the same direction from within 5 inches behind for at least 2 s, and engaging in anogenital interaction if the sniffing behavior occurred within 2 inches of the rear of the other animal. The number of seconds of each activity were expressed as a percentage of total test time.

#### 4.2.7 Three-chamber social novelty

Social preference and social memory were assessed using a 3-chambered box (60 × 24 cm total), with an empty central area separated on either side by Plexiglas from two areas containing removable wire cups. For the first 5 min of the test, the mouse was placed in the empty central area of the box with two removable plastic doors preventing movement into the side chambers. After 5 min of acclimation, a juvenile, male, stranger mouse was placed in one of the wire cups (alternating sides) and the doors were removed to allow the mouse to explore for 10 min. After 10 min, a new stranger was placed in the other wire cup and the mouse was allowed another 10 min to explore. Interactions were scored manually from video recordings by a researcher blinded to the experimental groups.

#### 4.2.8 Fear conditioning

Cued and contextual fear conditioning (FC) were used to evaluate fear-based learning and memory. Animals were tested as previously described (Weeber et al., 2002). Briefly, animals were trained in the fear conditioning apparatus with two 30 s acoustic conditioned stimuli (CS), each paired with an unconditioned stimulus (US): a 0.5 mA foot shock. To assess contextual fear conditioning, mice were placed back into the same context for 3 min, 24 h following the CS-US pairings. To assess cued fear conditioning, mice were placed in a novel context, allowed to acclimate for 3 min, then presented with the acoustic CS for 3 min. Tests were recorded, and fear response was assessed as freezing. Tests were scored by a researcher blinded to the experimental conditions, and the animal was considered to be freezing in the absence of movement with the exception of respiration.

#### 4.2.9 Six-arm radial arm water maze

We used a six-arm RAWM apparatus, with arms measuring 20 × 35 cm in a circular pool, with a 40 cm diameter center area. Escape platforms were removable clear Plexiglas, magnetically affixed to the end of each escape arm with ∼1 cm of water over the surface. Water was made opaque with white, non-toxic paint. Mice were trained in three trials per day for 4 days. For trial 1 the mouse was placed in the starting arm, which remained consistent throughout all tests, and was allowed to explore for 2 min or until it “escaped” by finding one of three available escape platforms. After 30 s to reference large, brightly colored spatial cues placed around the pool, the mouse was removed, gently dried, and placed in a warmed holding cage for 2 min. For trial 2, the process was repeated with the escape platform used in trial 1 removed, and this process repeated for trial 3. Testing was timed and scored in real time by a researcher blinded to the experimental groups. Spatial memory errors were considered entries into any arm that never contained a platform and working memory errors were considered repeated entry into any arm. A recorded entry required all four paws inside the arm of the maze. Testing continued until all groups reached the final platform with an average of <20 s.

#### 4.2.10 Y-maze

We measured spontaneous alternation task in the Y-maze apparatus, which consisted of three arms (38 × 8 cm) connected at 120° angles from each other, evenly lit from above. Mice were allowed to explore for 8 min, starting in one arm of the maze. Total entries and order of entries were recorded.

### 4.3 Diazepam treatment

A separate cohort of mice that had not previously been exposed to the OF apparatus were acclimated to handling for 3 days prior to treatment. Mice were treated with 1.5 mg/kg diazepam (7-chloro-1-methyl-5-phenyl-3H-1,4-benzodiazepin-2(1H)-one) (Sigma), diluted in sesame oil (Sigma) via subcutaneous injection, or sesame oil only. Mice were placed into an individual standard cage and transported to the behavioral room containing the OF. Mice were placed in the OF and allowed to explore for 45 min, and analysis was split into 15-min bins.

### 4.4 Western blotting

Mice were briefly anesthetized using isoflurane and sacrificed by rapid decapitation. Brains were dissected immediately to collect hippocampus, cortex, and PFC, and snap frozen on dry ice.

Western blotting was performed as previously described (Stevanovic et al., 2017). Tissue lysis was achieved by sonication in Mammalian Protein Extraction Reagent (M-PER) with phosphatase and protease inhibitors (10 μl/ml, Halt™ Protease and Phosphatase Inhibitor Cocktail, Thermo Scientific Inc., Rockford, IL, USA) clarified by centrifugation at 20,000 *g* at 4°C for 20 min after a 15 min incubation. Total protein concentrations were determined by BCA assay (Thermo Scientific), and equal amounts of protein were resolved on 9% tris-glycine acrylamide gels. Proteins were transferred for 1 h on ice, and nitrocellulose membranes were blocked in 5% milk in tris buffered saline with 0.1% tween-20 (TBS-T) before primary antibody incubation. When blots were probed for multiple proteins of different sizes, membranes were stained with Ponceau stain before blocking and cut. Membranes were incubated in 2% milk in TBS-T with primary antibody overnight, then rinsed in TBS-T and incubated in 2% milk in TBS-T with 1:7,500 HRP-conjugated secondary antibody for 2 h at room temperature. Rinsed blots were imaged with an image analyzer (Amersham Imager 6000), visualizing bands using enhanced chemiluminescence (ECL, Thermo scientific). Densitometry was performed with Image Lab (Bio-Rad Laboratories, Hercules, CA, USA), and bands of proteins of interest were normalized to beta-actin.

### 4.5 Immunohistochemistry and analyses

A subset of mice from each group was deeply anesthetized with sodium pentobarbital or isoflurane and transcardially perfused with saline, followed by 4% paraformaldehyde. Brains were post-fixed in 4% formaldehyde for 48 h at 4°C, cryoprotected in 30% sucrose in phosphate-buffered saline (PBS), and then sectioned coronally on a vibratome or sliding microtome at a thickness of 40 μm.

#### 4.5.1 Immunofluorescent staining

We visualized fast-spiking interneurons in the PFC using immunofluorescent staining for parvalbumin (PV), and inhibitory cells using GAD67 as a marker. Three to four sections containing PFC were selected and rinsed in PBS. Antigen retrieval was performed in 2N HCL for 1 h at RT, followed by tissue permeabilization in 0.1% triton-x in PBS. Tissue was blocked for 1 h at RT in 5% normal goat serum with 0.1% bovine serum albumin and 0.3% triton-x in PBS. This solution was used as the antibody dilution solution (ADS). Sections were incubated overnight at 4°C in primary antibodies against PV (1:1,000, Sigma-Aldrich, Cat# P3088, RRID:AB_477329) and GAD67 (1:500, Sigma-Aldrich, Cat# G5419, RRID:AB_261978) in ADS. Sections were protected against light from this point forward. After thorough rinsing in PBS, sections were incubated in goat anti-mouse iGg1 Alexa fluor 488 (1:500, Thermo Fisher Scientific, Cat# A-21121, RRID:AB_2535764) and goat anti-mouse iGg2a Alexa fluor 594 (1:500 Thermo Fisher Scientific, Cat# A-21135, RRID:AB_2535774) for 2 h at RT in ADS.

We visualized adult-born cells and mature neurons in the hippocampus using immunofluorescent staining for BrdU and NeuN. We selected six sections containing hippocampus at a 480 μm interval, beginning with the most rostral hippocampal section. We used the same immunofluorescence procedure outlined above, using primary antibodies raised against BrdU (1:250, Cell Signaling Technology, Cat# 5292, RRID:AB_10548898) and NeuN (1:250, Cell Signaling Technology, Cat# 12943, RRID:AB_2630395) and goat anti-mouse Alexa fluor 488 and goat anti-rabbit Alexa fluor 594 secondary antibodies (cited above).

Sections were rinsed in PBS, mounted on charged slides, and allowed to dry for 30 min. When dry, sections were rinsed with Milli-Q water, allowed to dry, and coverslipped with Prolong gold antifade reagent with DAPI.

#### 4.5.2 Fluorescent imaging and cell counting

Imaging for the 3-day post-injection BrdU study was done using a Zeiss AxioScan.Z1 slide scanner to capture stitched digital images of whole sections. BrdU+ cells were identified by hand by position and morphology and counted exhaustively in the entire DG.

PV/GAD67 and BrdU/NeuN images were acquired using a Zeiss LSM 880 confocal laser scanning microscope using equal laser intensity and exposure between compared images. Cells were counted in ImageJ by creating merged, maximum intensity z-stack projections. Colocalization was confirmed in Zen Blue 3.2 by using orthographic z-stack views.

#### 4.5.3 Doublecortin

We visualized immature neurons using doublecortin (DCX). DCX immunohistochemistry was performed as previously described (Lussier et al., 2013). Briefly, antigen retrieval was performed on free-floating tissue sections by incubation in sodium citrate (6.0 pH, 95°C, 30 min). Sections were then incubated with rabbit anti-DCX monoclonal antibody (1:1,000; diluted in 5% NGS, 1% BSA, 0.5% Triton-x 100 in PBS, 24 h). Day 2, sections were incubated in H_2_O_2_ (5%, 30 min), followed by biotinylated secondary antibody (goat anti-rabbit, Southern Biotech, 1:500, diluted as above, 1 h), and an avidin-biotin peroxidase complex (1:500, Vector Labs, 1 h). Immunolabeling was visualized with 0.025% diaminobenzidine, 4.167% nickel ammonium sulfate, and 0.002% H_2_O_2_ diluted in 0.175 M sodium acetate. The reaction was stopped using 0.175 M sodium acetate as a rinse. The sections were rinsed in PBS, mounted, and cover slipped using DPX mountant.

DCX+ cell number was quantified using unbiased stereology in Stereo Investigator 11.03 (MBF Bioscience, Williston, VT, USA), at 400× on a (microscope info) brightfield microscope equipped with a motorized stage (company info). DCX+ cells were counted using the optical fractionator method (West et al., 1991). The subgranular zone and granule cell layer in both hemispheres were selected as the region of interest and traced at 100× closely following cell bodies in five sections per subject, at an evaluation interval of 12. A mounted thickness of 20 μm was used, with 2 μm guard zones at the top and bottom of each section. A sampling grid size of 150 μm × 150 μm was placed randomly over the ROI to position the 50 μm × 50 μm counting frame in xy-axis. Approximately 200–300 cells were counted per subject, and the total number of cells calculated using the following formula Ntotal: Σ*Q*- × *t*/*h* × 1/asf × 1/ssf, where Q- is the number of DCX+ cells counted, *t* is the mounted thickness of the sections, *h* is the counting frame height, asf is the area sampling fraction, and ssf is the section sampling fraction (West et al., 1991; Gundersen et al., 1999).

Characterization of immature neurons in the DG was performed to produce a semiquantitative description of DCX+ cell development. The dendritic branching of all cells within the stereological counting frame were categorized as previously described (Plümpe et al., 2006; Lussier et al., 2011). Each cell was categorized as 1–6 based on the extent of its dendritic processes. Categories 1 and 2 defined cells in the proliferative stage with no processes, or one short process. Categories 3 and 4 defined intermediate cells with one medium process or one process reaching into the molecular layer. Categories 5–6 defined cells in the postmitotic stage with one secondary dendritic branch in the molecular layer, or a delicate, well-defined dendritic tree.

#### 4.5.4 Golgi impregnation, dendritic spine counting, and characterization

We used Golgi-Cox impregnation to visualize dendritic spines in a subset of neurons, allowing us to count and characterize spines in the hippocampus. We deeply anesthetized four mice per group with isoflurane and performed rapid decapitations. Brains were cut along the midline, and both hemispheres were rinsed in Milli-Q water and placed immediately in impregnation solutions. Golgi-Cox impregnation was performed according to protocol using the FD Rapid GolgiStain Kit (FD Neurotechnologies). Briefly, tissue was stored in the dark in impregnation solution for 3 weeks and gently agitated daily, then moved to Solution C. Tissue was rapidly frozen in dry-ice cooled isopentane and stored at −80°C until sectioned at 100 μm using a cryostat and mounted on gelatin-coated slides. Mounted tissue was stained using freshly mixed Solution D/E, dehydrated in an ethanol series, and cover slipped using Permount mounting medium.

Slides were imaged using a 60× Uplan FL N 1.25 NA Oil Iris objective on an Olympus BX53 digital microscope. Primary basal dendrites and secondary apical dendrites were imaged from hippocampal pyramidal cells. Secondary dendrites were imaged from DGC. Dendritic spines were counted manually from z-stack images of various depth, along an approximately 20 μm length of the dendrite of interest, starting at least 5 μm from any branch point. The actual length of the counted segment was measured and used to generate the linear density.

### 4.6 Statistical analysis

One-way ANOVA was used to determine group differences unless otherwise indicated in the text. Significant main effects of genotype were further assessed using LSD *post-hoc* tests. Levene’s test of homogeneity of variances was used, and if significant, Welch’s test of equality of means was used in place of ANOVA to determine group differences, followed by *post-hoc* testing using the Games–Howell correction. Repeated measures ANOVA was used to evaluate group differences in Rotarod, RAWM, FC, FST, and DCX+ cell categorization. Poisson regression and Wald Chi-square analysis was used to further analyze RAWM results. FST was further analyzed by an independent-samples Kruskal–Wallis test with *post-hoc* testing with the Dunn–Bonferroni correction. Independent samples two-tailed *t*-tests were used to analyze GABA receptor, PV/GAD67, BrdU, and NeuN expression results, with t statistics reported as absolute values and direction of change indicated in the text. Outliers were excluded by data point if values were outside 2 SDs from the group mean unless otherwise indicated. The criterion for statistical significance was set at *p* < 0.05. Statistics were performed using IBM SPSS Statistics 22 (SPSS, Inc., Chicago, IL, USA). Data are presented as mean ± SEM, unless otherwise indicated in the text.

## Data availability statement

The datasets presented in this study can be found in online repositories. The names of the repository/repositories and accession number(s) can be found below: https://doi.org/10.7910/DVN/FH1XKN, Harvard Dataverse.

## Ethics statement

The animal study was approved by the University of South Florida Institutional Animal Care and Use Committee. The study was conducted in accordance with the local legislation and institutional requirements.

## Author contributions

HM: Conceptualization, Data curation, Formal analysis, Funding acquisition, Investigation, Methodology, Visualization, Writing – original draft, Writing – review & editing. CB: Investigation, Visualization, Writing – review & editing. HJ: Investigation, Writing – review & editing. BC: Investigation, Visualization, Writing – review & editing. CM: Investigation, Validation, Writing – review & editing. DGo: Investigation, Writing – review & editing. ES: Investigation, Writing – review & editing. JF: Investigation, Writing – review & editing. SR: Investigation, Writing – review & editing. AV: Investigation, Writing – review & editing. AO: Investigation, Writing – review & editing. BC: Investigation, Writing – review & editing. AL: Conceptualization, Investigation, Methodology, Writing – review & editing. LD: Writing – review & editing. EW: Conceptualization, Writing – review & editing. JG: Conceptualization, Writing – review & editing. DGu: Conceptualization, Formal analysis, Funding acquisition, Resources, Supervision, Visualization, Writing – original draft, Writing – review & editing.
